# Conceptual and methodological challenges for neuroimaging studies of autistic spectrum disorders

**DOI:** 10.1186/1744-9081-6-17

**Published:** 2010-03-09

**Authors:** Luigi Mazzone, Paolo Curatolo

**Affiliations:** 1 Division of Child Neurology and Psychiatry, Department of Pediatrics, University of Catania, Catania, Italy; 2 Tor Vergata University of Rome, Rome, Italy

## Abstract

Autistic Spectrum Disorders (ASDs) are a set of complex developmental disabilities defined by impairment in social interaction and communication, as well as by restricted interests or repetitive behaviors. Neuroimaging studies have substantially advanced our understanding of the neural mechanisms that underlie the core symptoms of ASDs. Nevertheless, a number of challenges still remain in the application of neuroimaging techniques to the study of ASDs. We review three major conceptual and methodological challenges that complicate the interpretation of findings from neuroimaging studies in ASDs, and that future imaging studies should address through improved designs. These include: (1) identification and implementation of tasks that more specifically target the neural processes of interest, while avoiding the confusion that the symptoms of ASD may impose on both the performance of the task and the detection of brain activations; (2) the inconsistency that disease heterogeneity in persons with ASD can generate on research findings, particularly heterogeneity of symptoms, symptom severity, differences in IQ, total brain volume, and psychiatric comorbidity; and (3) the problems with interpretation of findings from cross-sectional studies of persons with ASD across differing age groups. Failure to address these challenges will continue to hinder our ability to distinguish findings that outline the causes of ASDs from brain processes that represent downstream or compensatory responses to the presence of the disease. Here we propose strategies to address these issues: 1) the use of simple and elementary tasks, that are easier to understand for autistic subjects; 2) the scanning of a more homogenous group of persons with ASDs, preferably at younger age; 3) the performance of longitudinal studies, that may provide more straight forward and reliable results. We believe that this would allow for a better understanding of both the central pathogenic processes and the compensatory responses in the brain of persons suffering from ASDs.

## Background

Autistic Spectrum Disorders (ASDs) are a class of conditions that embodies Autistic Disorder, Asperger's syndrome, and Pervasive Developmental Disorder Not Otherwise Specified (PDD-NOS). Each of these conditions is defined by the presence of complex developmental disabilities that include qualitative impairments in social interactions (i.e. impaired use of non-verbal behaviors, failure to develop peer relationships, and poor social reciprocity) and in communication (i.e., delay in development of spoken language, inability to sustain a spoken conversation, stereotyped use of language, paucity of symbolic or imitative play), and restricted or stereotyped interests and behaviors. Persons with ASDs must manifest symptoms by the age of three years. Intelligence Quotients (IQ) vary widely, but the overall prevalence of intellectual disability in this population is around 50-75% [1].

Recent years have witnessed innovative approaches to the study of ASDs, driven by the emergence of new technologies and methodologies for studying both normal and pathological development in children and adolescents. Among these, some of the most important techniques include anatomical magnetic resonance imaging (MRI) and functional MRI (fMRI), which can reveal anatomical and functional abnormalities in brain development. Both of these MRI modalities have played a major role in advancing our knowledge of the neural bases of ASDs [2].

Anatomical studies have documented in persons with ASDs increases in brain volumes, particularly in the posterior regions, and especially in the right hemisphere [3-7]. These observations are consistent with well-replicated findings of enlarged head circumference in very young autistic children as compared to healthy children, that seems to derive from accelerated head growth at 6-14 months of age, despite normal or smaller head circumference at the time of birth [8-12]. Additional anatomical imaging studies have reported larger volumes of both white and gray matter in the frontal cortex [13], larger volumes of the caudate nucleus even after covarying for overall brain size [14,15], and abnormal volumes (usually larger) of the amygdala and hippocampus [16,17]. Finally, both smaller [18] and larger volumes [19,20] of the cerebellum have been reported.

Besides neuroanatomical studies, a large number of functional neuroimaging studies, designed to investigate the neural mechanisms underlying the core symptoms of ASDs, have recently been published.

Despite the great contribution that these imaging technologies have provided to the knowledge of morphologycal features in persons with ASDs, the unique phenotypic trademarks of these disorders create peculiar difficulties in designing imaging studies that provide data with univocal interpretation and new insight into the pathogenesis of these conditions. Some of these include: the difficulty that persons with ASDs have in performing the tasks used in fMRI studies in a way that is comparable to normal subjects; the heterogeneity in persons with ASD who are enrolled in research studies; and the problematic interpretation of findings from cross-sectional studies of persons with ASDs who belong to differing age groups.

Gaining a better understanding of the nature of the alterations in brain functions that occur in ASD could be of vital importance to try to identify the link between anatomical and functional abnormalities in the brain of persons with ASDs and their behavioral phenotypes. Our intent is to review in a critical way the knowledge accomplished so far in functional imaging studies of ASDs as well as to identify the gaps that still need to be addressed, trying to overcome the problems and challenges that may have limited our knowledge on the pathogenesis of this type of disorders.

### fMRI task design

Several fMRI studies, many published in the last decade, used specifically designed tasks to investigate abnormalities in discrete neural systems in persons with ASDs. In particular, some studies have investigated the role played by specific perceptual, cognitive and attentional processes underlying the executive functions [21,22]. Other studies used specific tasks for language-comprehension to investigate functional connectivity and semantic processes [23,24]. Finally, a great number of studies used tasks involving face perception or emotional processes (table 1) to identify brain disturbances that might account for the profound impairments, typical of persons with ASDs, to interact socially and to recognize the emotions of others. Findings from these studies, however, have been inconsistent and often contradictory. The inconsistencies across studies likely derive from the difficulty that persons with ASDs have with processing strategies, and from the degree of arousal, effort, frustration, or confusion that they manifest while performing the task. This could represent one of the reasons why persons with ASDs turn out in showing such a variable brain activity during functional imaging tasks. Moreover, the activations associated with these differences could be linked to epiphenomenal effects that are associated with differing performance levels across groups on a given task. Finally, it would probably be useful to select an appropriate control task suitable for the primary task of interest.

**Table 1:** MRI studies published between 2005 and 2009 exploring *facial processing *in samples of at least 10 patients with ASD, as determined by a PubMed search

STUDY	AUTISM GROUP *N Sex* *Age (M ± SD)*	CONTROL GROUP *N Sex* *Age (M ± SD)*	TASK DESIGN *[Cognitive process]*	RESULTS IN ASD GROUP
Welchew DE et al., 2005[64]	13 M (31.2 ± 9.1)	13 M (25.6 ± 5.1)	Faces expressing different intensities of fear [Emotional cognitive process]	Abnormal functional connectivity of medial TL

Dalton KM et al., 2005[38]				
*Study I* *Study II*	14 M (15.9 ± 4.71) 16 M (14.5 ± 4.60)	12 M (17.1 ± 2.78) 16 M (14.5 ± 4.56)	Facial emotion discrimination task [Emotional cognitive process] Facial recognition task [Cognitive perception]	Activation in the FG and AMY strongly and positively correlated with the time spent fixating the eyes in both studies

Dapretto M et al., 2006[65]	9 M 1 F (12.05 ± 2.5)	9 M 1 F (12.38 ± 2.2)	Face emotional recognition task [Emotional cognitive process]	No activation in the IFG

Bölte S. et al., 2006[66]	5 M (29.4 ± 5.9)	5 M^a^ (25.8 ± 8.0)	Face recognition pre and post after FEFA [Emotional cognitive process]	No significant activation changes in the FG pre- and post-training

Bird G. et al., 2006[67]	14 M 2 F (33.3 ± 11.5)	14 M 2 F (35.3 ± 12.1)	Task in which pairs of face and house stimuli were present on every trial [Social and cognitive perception]	Failure of attention to modulate connectivity between extra striate areas and V1

Wang AT et al., 2007[68]	18 M (12.5 ± 2.9)	18 M (11.8 ± 1.9)	Irony comprehension [Social and cognitive perception]	Reduced activity in the medial PFC and right STG

Ashwin C. et al., 2007[69]	13 M (31.2 ± 9.1)	13 M (25.6 ± 5.1)	Perception of fearful faces [Emotional cognitive process]	Increase in the ACC and STC

Hadjikhani N.et al., 2007[70]	8 M 2 F (34 ± 11)	4 M 3 F (35 ± 12)	Passively viewing non emotional faces [Cognitive perception]	Significant activation of FG and IOG; Hypoactivation in right AMY, IFC, STS, and face-related somatosensory and premotor cortex

Dichter GS et al., 2007[71]	16 M 1 F (22.9 ± 5.2)	14 M 1 F (24.6 ± 6.5)	Reaction time to arrow or gaze stimulus with similar flanker stimuli oriented (congruent or incongruent directions) [Cognitive perception and control]	Hypoactivation in MFG, right IFG, bilateral intraparietal sulcus, and the ACC during incongruent gaze stimuli

Koshino H. et al., 2008[72]	11 M (24.5 ± 10.2)	10 M 1 F (28.7 ± 10.9)	*n*-back working memory task involving face recognition [Working memory]	Hypoactivation in the left IPFC and in the right PTC

Kleinhans NM et al., 2008[73]	19 (23.5 ± 7.8)	21 (25.1 ± 7.6)	Facial emotion discrimination (familiar, unfamiliar and new friend) [Emotional cognitive process]	No between-group differences in fusiform activation to faces or houses; Significant FG-AMY and FG-STS functional connectivity

Pinkham AE et al., 2008[74]	12 M (24.1 ± 5.7)	12 M^b ^(27.1 ± 3.9) 12 M^c ^(26.4 ± 5.2) 12 M^d ^(28.0 ± 3.9)	Complex social judgments of faces [Social and cognitive perception]	Reduced activation in the right AMY, FG, VLPFC

Humphreys K. et al., 2008[75]	13 M (27 ± 10)	15 M (29 ± 10)	1) Conventional face and object mapping 2) Motion pictures experiment [Emotional cognitive process and perception]	Decreased of activation, not only in fusiform face area but also in STC and occipital area

Uddin LQ et al., 2008[76]	12 M (13.19 ± 2.6)	12 M (12.23 ± 2.10)	Responsiveness to images of the subjects' own face and of others' faces [Emotional perception]	Activation of right PM/PF system while viewing images containing mostly their own face

Bookheimer SY et al., 2008[77]	12 M (11.3 ± 4.0)	12 M (11.9 ± 2.4)	Subjects had to match faces presented in the upright versus and inverted position [Cognitive perception]	No differences in the FG; Decrease in activation in left PFC; No activation in AMY for upright task

Pierce K. et al., 2008[78]	9 M 2 F (9.9)	9 M 2 F (9.8)	Pictures of a familiar adult or child, stranger adult or child, objects [Emotional cognitive process and perception]	Deficit in the mean number of significantly active voxels in FG looking at stranger adults face

Corbett BA et al., 2009[79]	12 (8-12)^e^	15 (8-12)^e^	Matching facial expressions and people [Emotional cognitive process and perception]	Reduction of FG and AMY activation involved in face processing

(Key words: "ASD and fMRI", "Autism and fMRI", "Asperger and fMRI", "High-functioning Autism and fMRI" High-functioning ASD and fMRI", "Autism and fMRI and facial processing").

TL = temporal lobe; FG = fusiform gyrus; AMY = amygdala; Frankfurt Test and Training of Facial Affect Recognition (FEFA); V1 = primary visual cortex; PFC = prefontal cortex; STG = superior temporal gyrus; ACC = anterior cingulate cortex; STC = superior temporal cortex; IOG = inferior occipital gyrus; IFC = inferior frontal cortex; STS = superior temporal sulcus; MFG: midfrontal gyrus; IFG = inferior frontal gyrus; IPFC = inferior prefrontal cortex; PTC = posterior temporal cortex; VLPFC = ventrolateral prefrontal cortex;PM premotor; PF = prefrontal.

^a^This five participants were ASD and were not randomly assigned to the experimental group for receiving emotion recognition training.

^b^normal control.

^c^individuals with schizophrenia or schizoaffective disorder with prominent paranoid symptoms.

^d^individuals with schizophrenia or schizoaffective disorder without paranoid symptoms.

^e ^Age range

#### An example: face perception tasks

Many behavioral studies have consistently shown that persons with ASDs are selectively impaired in their ability to recognize faces [25-33]. In addition to this, recent fMRI studies have shown that this selective impairment is associated with abnormal patterns of brain activation. Most of these studies consistently reported an atypical pattern of activation in the fusiform gyrus, which is extensively activated during face processing in healthy individuals but seems to be much less activated during the same tasks in individuals with ASDs [34,35]. One of the first neuroimaging studies of face processing in individuals with ASDs, reported decreased activation in the fusiform gyrus during face discrimination as compared to control subjects [36]. This study also reported that persons with ASDs activate object-processing regions, such as the right inferior temporal gyrus, when viewing faces. However, a recent clinical study showed that children with ASDs spend more time looking at an adult's mouth instead of gazing into the eye [37] and many other clinical studies have used eye tracker to detect what participants were looking at during the face discrimination task. Nevertheless, before 2005, no fMRI study had reported on the relationship between the direction and duration of gaze fixation and the patterns of brain activation during the processing of human faces in individuals with ASDs. The first study to address this point [38], showed that the activation of the fusiform gyrus during face perception in ASD patients correlated positively and strongly with the duration of the participants fixation on the eye region of the face being viewed. In addition, this study reported that ASD participants spend less time than control subjects looking at the eyes of faces during face perception tasks, focusing instead on other, isolated features of the face, such as the mouth. Thus, the failure to fixate on the eyes may be the proximal cause for reduced activation of the fusiform gyrus in patients with ASD during face perception tasks. In other words, the commonly reported hypoactivation in the fusiform gyrus during face processing in ASDs may be due not necessarily to an abnormality in the fusiform gyrus activity itself, but rather to the way in which individuals with ASDs scan the face during the fMRI task. This highlights a key difficulty when dealing with the interpretation of fMRI findings in ASDs: findings of hypo- or hyper-activation in a given area do not per se license the inference that that area is somehow activated abnormally. Their tendency to focus on the details of the face being presented rather than on the overall face may also explain the activation of the infero-temporal gyrus, a region that is responsible for object perception, during face perception tasks, instead of activating the fusiform gyrus. Furthermore, differences in activation between autistic and healthy children may reflect differences in attentional behavior, rather than differences in activation for any given task across groups.

Different fMRI studies that used tasks based on the recognition of faces actually employed various task conditions, including a passive or active viewing of faces [39], the explicit discrimination of gender in pictures of familiar and unfamiliar faces [40], and the explicit labelling of facial expressions of emotion [41,42]. These tasks involving the perception of faces imply different perceptual strategies among subjects that result from the different degree of familiarity or level of "expertise" that the person has with specific stimuli. Therefore, differences in activation in face area may also represent differences in task processing strategies or in the level of "expertise" of the subjects. This peculiarity in performing the task could thereby produce group differences in brain activation that are not entirely valid or reliable.

Differences in activation may also arise from difficulties in comprehending the significance of the scan session that can generate confusion or anxiety during the performance of the task, as well as from epiphenomenal features that could be associated with differing performance levels on the task across groups (e.g. emotional and cognitive reactions to recognition of performing poorly). The point is that all of these situations are likely to differ systematically in the ASDs group compared with typically developing controls, and therefore group differences in activation could reasonably derive from these contextual factors rather than from the cognitive process that is explicitly being studied.

To summarize, the variations in the activity of the fusiform area during face tasks could be influenced by several confounding factors, including the duration of gaze fixation, the emotional valence and arousal induced by the stimulus and the level of expertise and familiarity with the stimulus. For all these reasons autistic persons do not show the same facial features as non autistic persons and it is not surprising that the fusiform gyrus does not activate during facial recognition tasks in ASDs. In conclusion very little can be learnt from a tasks that subjects cannot perform adequately.

### Influence of the heterogeneity of samples on studies of ASDs

Another challenge when designing an fMRI study on ASDs is the widely acknowledged difficulty of recruiting a homogeneous group of patients. This difficulty stems from the number of variables that ideally should be accounted for within a sample of persons with ASDs, including the broad range of symptoms, the chronicity of illness, differences in IQ, and psychiatric comorbidity. Considering that the differences between instances of ASD can be extreme, the comparison between participants in a single study as well as across different studies is difficult and could be unreliable, thus compromising the strength of the findings. The majority of fMRI studies with autistic participants involve both adolescents and adults, by reason of the difficulty in scanning younger patients and in obtaining suitable age-matched controls. However, adolescents and adults may differ significantly from one another in terms of symptoms, compensatory responses, and brain structure and function, due to the chronicity of the illnesses itself and to the effects that the chronic severe illness can have on their brain.

Persons with ASDs can manifest various cognitive abilities and in several prior studies, this has produced confounding findings and non-univocal interpretations. Early studies of autism reported reduced size of the cerebellum in living autistic persons, showing lobules VI and VII 19% smaller than normal controls [18], which seemed consistent with preliminary reports of reduced Purkinje and granule cell numbers in postmortem studies [43,44]. However, subsequent studies largely failed to replicate these initial findings [3,45]. These contradictory observations could be explained by the fact that the initial studies used a sample of children with autism that included participants with comorbid mental retardation, and thus the alteration of cerebellum volume could be linked with mental retardation more than with ASDs [4]. This suggests that the initial findings of cerebellar hypoplasia were most likely driven by mismatches in IQ across the ASD and control groups, and consequently represented a nonspecific result associated with mental retardation, instead of autism.

A recent approach to solve this problem has been to focus exclusively on participants who are either high-functioning or who have Asperger's syndrome and that, for this reason, are also better able to participate successfully in the scanning procedure than low-functioning persons. This solution, however, forces investigators to study only a small percentage of individuals with ASDs (15-20%), severely limiting the generalization of the findings to the broader population of individuals with ASDs.

Another important consideration in imaging studies on persons with ASDs is their peculiar heterogeneity in brain volume, as suggested by recent studies, according to which up to 10% of the subjects with ASDs could have an enlargement of the volume of the total brain [6,7,10], even if it is not completely clear whether this alteration persists during adolescence [46,47]. For this reason, studies measuring the volume of specific brain areas should covary the volume of individual brain regions for the total brain volume.

Several studies involving persons across a wide range of ages have reported the cerebellum to be enlarged in ASDs persons compared to normal controls [19,20]. However, the increase in cerebellum volume could also reflect the increase in the total brain volume. In support to this hypothesis, the only recent study that has examined very young children with ASDs (<3 ys) did not detect any alteration of cerebellum volume [10].

Finally, another crucial difference among ASDs persons is the presence of psychiatric and neurologic comorbid disorders that can introduce additional heterogeneity to the samples. Increased rates of anxiety disorders, depression, and obsessive-compulsive disorder have been identified in persons with ASDs since many years [48,49]. Epilepsy affects 35-40% of persons with ASD [50]. Comorbid psychiatric symptoms are difficult to recognize and diagnose in persons with ASDs because they can be obscured by the more prominent core symptoms of ASDs. Moreover, symptoms that are part of the ASD phenotype are also present in other disorders and can contribute to render the diagnosis more complicated. The repetitive thoughts and behaviors that are characteristic and disabling core features of ASDs, for example, may or may not index undetected comorbid obsessive-compulsive disorder. The difficulties in distinguishing between symptoms of ASDs and other diseases can complicate the interpretation of fMRI data, in the sense that it becomes difficult to determine which abnormal activation is associated with which disorder.

### Developmental correlates of a disease process: the need for longitudinal studies

A third serious challenge for the interpretation of findings from prior existing neuroimaging studies of ASDs is the absence of longitudinal studies that would help to define the true course of changes in brain structure and function that accompany changes in symptoms of ASD. So far, imaging studies of ASDs have all been cross-sectional and typically have investigated only adolescents and adults, in whom the core pathophysiological processes of ASDs are likely to be thoroughly entangled with the compensatory responses and with the effects that chronic illness and adverse life experiences have on the brain. Cross-sectional findings are useful for generating hypotheses for further testing, but these hypotheses need to be confirmed by longitudinal investigations. All imaging studies of neurological or psychiatric disorders face the challenge of distinguishing between findings that represent core pathophysiological processes and findings that are simply epiphenomena, representing compensatory or adaptive changes in the nervous system. Children suffering from ASDs can manifest a range of symptoms that prominently influence their sensory, cognitive, and emotional experiences beginning in their first years of life [51]. In turn, these peculiar experiences, that may be different from those encountered by typically developing children, could influence their brain development and induce compensatory systems that may alter both morphological volumes and regional activation.

Numerous cross-sectional studies that investigated volumes of the amygdala and hippocampus have generated contradictory findings [7,17,47,52-55]. In particular, studies of the amygdala, that has been proposed as one of the possible brain regions candidate to be responsible for the social deficit of ASDs, have shown increased [7,56], decreased [47,57], or normal amygdala volumes [58] (table 2). Undoubtedly the use of unreliable morphometric procedures contributed to breed these inconsistencies. However an additional explanation likely relates to the composition of the participant sample, being formed by differing age groups with differing degrees of remitting and non-remitting symptoms, as well as differing etiological subtypes. The role of amygdala has been investigated also by functional studies that found different degree of amygdala activation in response to different emotional and perceptional tasks (table 2).

**Table 2:** Cross sectional neuroimaging studies implicating the amygdala in Autism Spectrum Disorders

	Study	ASDs Group ***N Age (M ± SD)***	Ctr Group ***N Age (M ± SD)***	Results	
***Anatomical Studies***	Howard MA et al., 2000[56]	10 (15.8-40.3)^a^	10 (age matched)	Increased	
	
	Sparks BF et al., 2002[7]	45 (47.4 ± 4.2)^b^	26 (47.5 ± 6.2)^b^ 14^c ^(47.5 ± 5.6)^b^	Increased	
	
	Aylward EH et al., 1999[47]	14 (20.5 ± 1.8)	14 (20.3 ± 1.7)	Decreased	
	
	Pierce K. et al., 2001[57]	7 (21-41)^a^	8 (20-42)^a^	Decreased	
	
	Haznedar MM et al., 2000[58]	17 (27.7 ± 11.3)	17 (28.8 ± 9.4)	Normal	
	
	Schumann CM 2004[17]	71 (7.5 - 18.5)^a^	27 (7.5-18.5)^a^	Amygdala initially increased but does not undergo the age-related increase	
	
	Nacewicz BM et al., 2006 [55]	16 (14.3 ± 4.7)	14 (13.7 ± 3.9)	Decreased	
	
	Munson J. et al., 2006 [54]	45 (47.4 ± 4.2)^b^	**-**	Increase in right amygdala	
	
	Schumann CM et al., 2009 [80]	50 (22-61)^b^	39 (20-51)^b^	Increased	

				***Results***	**Task Design**

***Functional Studies***	Baron Cohen S. et al., 1999[81]	6 (26.3 ± 2.1)	12 (25.5 ± 2.8)	Lack to AMY activity when making mentalistic inferences from the eyes	Interfering mental status from the eyes region
	
	Critchley HD et al., 2000[82]	9 (37 ± 7)	9 (27 ± 7)	Failed to activate left AMY in the implicity task	Explicitly and implicitly processing emotional facial
	
	Wang AT et al., 2004[42]	12 (12.2 ± 4.8)	12 (11.8 ± 2.5)	AMY activity moderated by task demands in control but not in ASD	Face labelling vs matching emotional expression
	
	Ashwin C. et al., 2007[69]	13 (31.2 ± 9.1)	13 (25.6 ± 5.1)	Controls showed greater activation in the left AMY	Fearful face-processing
	
	Kleinhans NM et al., 2009[83]	19 (21.9 ± 5.9)	20 (24.7 ± 7.9)	AMY hyperarousal	Upright neutral faces, inverted neutral faces
	
	Corbett BA et al., 2009[79]	12 (8-12)^a^	15 (8-12)^a^	Diminished activation of the AMY in emotion matching	Matching facial expressions and people

^a^age range

^b^months

^c^idiopathic developmental delays (DD)

AMY = amygdala

Most of the neuroimaging studies of ASDs conducted so far have typically been cross-sectional, comparing one particular brain imaging measure at a single time point across samples. The findings from these studies are interpreted as representing an abnormality in the patient group compared with typically developing controls, which directly contributes to the symptoms and the disease process. One study, for example, used anatomical MRI to examine volumes of the amygdala and hippocampus in boys, aged 7.5-18.5 years, with ASD and age-matched normal controls. This study found an enlarged amygdala and hippocampus in children with autism, with or without mental retardation, compared with normal controls. These findings were interpreted as follows: hippocampus volumes are probably larger at all ages whereas amygdala volumes are larger in young children, but not in adolescents [17]. However, this study was not adequately controlled for overall brain size, and the interpretation of the findings of developmental trajectories was based on cross-sectional data. In fact, considering that amygdala enlargement could be a marker of symptoms remission, remitted subjects are progressively excluded from the study because they show fewer symptoms. By contrast, smaller amygdala may exacerbate the autistic symptoms, making subjects at younger ages preferentially recruited in the study. For these reasons the interpretation of these findings could result from the ascertainment bias of studying only patients who were still symptomatic rather than a more representative sample of patients including also subjects with remitting symptoms. Another study of 45 children with ASD (without controls) reported an enlargement of right amygdala at 3 and 4 years of age that was associated with more severe baseline symptoms and with a worse clinical outcome at the age of 6 years [54]. Also in this case a larger amygdala exacerbates symptoms at baseline, because more severely affected subjects with larger amygdala were preferentially recruited at baseline compared with adolescents. A recent longitudinal study conducted on children between 2 and 4 years of age, shows an enlargement of the amygdala at 2 years in ASDs subjects compared to controls. However no relative increase in magnitude was observed between 2 and 4 years of age [59].

This confirmed that longitudinal studies are necessary to assess the developmental correlates of the disease process in ASDs in terms of differential growth of specific neural regions, as well as of the impact of the differing life experiences on brain development. However, during the course of the years, although the need for longitudinal studies has been outlined by some researchers [7], apparently a lot of difficulties still persist in performing this kind of studies. This could be due to several reasons, including the difficulties in scanning very young, low-functioning autistic children, as well as the challenges in recruiting individuals who are willing to participate in an experiment over an extended period of time.

### Recommendations

Recent advances in neuroimaging methodologies have undoubtedly helped to address some of the challenges in the study of ASDs. However if our aim is to better define the neural networks that underlie ASDs, several crucial issues remain unclear, and in this review we have tried to discuss some of them. In order to define the cause and effect and the developmental correlates of the disease process using neuroimaging, we will need to tackle the challenges of task design, heterogeneity of participant samples, and the absence of longitudinal studies.

The tasks used in fMRI paradigms should be simple enough in the design to minimize differences across groups in terms of effort, performance, and task processing strategies. The use of elementary tasks to demonstrate similar activations across age or diagnostic groups will help to reduce misleading interpretations and to understand where in the information processing mechanisms the differences in brain activation across ages or diagnoses first arise. The design of simple and passive tasks to examine sensory-perceptual functions may be also useful to study samples of lower functioning participants with ASDs. In addition to a simpler and more clearly targeted design for fMRI tasks, we should also take into account, especially dealing with infants and toddler for the study of early development of high-risk individuals, the use of resting perfusion studies. These studies represent perhaps the most useful approach to developmental investigations because they do not require the performance of a task and therefore issues of differing performance and changing strategies across ages are not relevant. The use of perfusion studies would also avoid the limitations of using particular behavioral tasks and their associated theoretical frameworks when studying brain functions in persons with ASDs. However, we would like to point out that the feasibility of conducting these studies is easier in Europe than in the U.S., considering that the U.S Institutional Review Boards in the past have frowned upon the use of radioactive isotope scans in research with young children.

Future studies of ASDs should ideally try to be performed on samples in which participants are matched across groups by IQ, age and gender, and recognizing the main comorbidities. A strategy that has recently been developed and that could help to minimize the issues related to the heterogeneity of the sample is the idea of "data sharing" proposed by Belmonte et al. [60]. Indeed, sharing data between different laboratories, although it needs to be carefully standardized, at least regarding the derived data, could contribute, by increasing the numerosity of subjects involved, and therefore of the data collected, the biases due to the heterogeneity of samples. Another big effort that has been done in the last few years in this direction is the attempt to develop some standardized measures such as Autism Diagnostic Interview-Revised (ADI-R) [61] and Autism Diagnostic Observation Schedule (ADOS)[62], in order to minimize the discrepancies into ASD group.

However, a crucial point for the ASD research community is the need to develop a better definition of what constitutes ASD syndromes and of which symptoms are present in each individual who is affected by an ASD. By diagnosing comorbid disorders and defining ASDs more clearly, we will be able to distinguish with greater accuracy the symptoms of comorbidities from the core symptoms of the primary disorder. Until symptoms of ASDs and comorbid disorders will not be defined more precisely, researchers and clinicians will have difficulties in delineating clearly the nature and scope of other psychopathologies as they covary with ASD. In turn, this lack of details and certainty in the definition of symptoms will continue to undermine our ability to study ASDs with imaging technologies.

Studies of ASDs should include progressively younger age groups, as well as high-risk cohorts prior to onset of illness [63], both of which will help to identify trait markers within the functioning of the central nervous system that predispose individuals to these illnesses. Of course it is not easy to scan very young children even if in a recent study the authors tried the strategy of performing MRI on children during natural nocturnal sleep in the evening after the child's normal bedtime or while the child was awake and watching a video, in order to try and keep the children more quiet [59]. Identifying trait markers in turn will help to identify differing subtypes of ASDs. By identifying trait markers and subtypes, we will then be able to design tasks that target functional systems with increased validity and specificity. Defining disease subtypes more clearly based on trait markers will in turn also help to identify disease features in the brain that represent compensatory responses to the presence of these disorders or the effects of medications used for specific treatment. Indeed trait markers can be followed longitudinally, before, during, and after the onset, to disentangle trait, state, and compensatory effects.

Future studies should also use samples that are epidemiologically ascertained within both cross-sectional and longitudinal frameworks. This will provide data that are more valid for inferences of the natural history and developmental correlates of ASDs than are data acquired in samples affected by ascertainment biases. Although imaging young persons with ASDs is challenging methodologically, longitudinal studies that begin as early as a diagnosis can be established are very helpful to clarify the relationships between developmental abnormalities in specific brain structures and functional deficits in autistic persons, because images acquired closer to the age of onset of the illness will minimize the effects of chronic illness and compensatory responses in the brain.

Finally, another issue to be taken into account is the fact that persons with ASDs almost certainly do not activate their brains in ways similar to unaffected controls during the performance of cognitive, affective, or behavioral tasks. Therefore, group differences in brain activation on any task likely tell us only limited informations regarding the pathophysiology of ASDs. Unfortunately, tasks on which persons with ASDs would activate normally are difficult to imagine, even if developing such a task in which these persons would be able to react in a way that is close to the behavior of non-autistic persons, thus identifying some areas that are activated normally in autistic subjects, would probably provide even more informations about the functioning of their neural system than will reports of group differences between ASDs and healthy control subjects.

Despite the many challenges that we have outlined in this review, the neuroimaging field continues to provide an increasingly important contribution to the understanding of the etiology and pathophysiology of ASDs, being a crucial tool that could help giving insights on the functions and development of the brain networks involved.

## Competing interests

The authors declare that they have no competing interests.

## Authors' contributions

LM overviewed the literature, pulled all the informations together and wrote the manuscript; PC contributed to theoretical interpretation and final proof reading. Each author read and approved the final version of the manuscript.
